# A blueprint for success: lessons learned from developing the official guide to Iranian undergraduate medical education accreditation

**DOI:** 10.1186/s12909-024-05215-6

**Published:** 2024-03-07

**Authors:** Mahdi Aghabagheri, Babak Sabet, Abtin Heidarzadeh, Ebrahim Kalantar, Ali Norouzi, Maryam Alizadeh

**Affiliations:** 1 Philosophy of Science Department, School of Medicine, Shahid Sadoughi University of Medical Sciences, Yazd, Iran; 2 Department of Surgery, Faculty of Medicine, Shahid Beheshti University of Medical Sciences, Tehran, Iran; 3 Medical Education Research Center, Guilan University of Medical Sciences, Guilan, Iran; 4 Smart University of Medical Sciences, Tehran, Iran; 5 School of Medicine, Education Development Center (EDC) and Social Determinants of Health Research Center, Zanjan University of Medical Sciences, Zanjan, Iran; 6 Department of Medical Education, School of Medicine and Health Professions Education Research Center, Tehran University of Medical Sciences (TUMS), Tehran, Iran

**Keywords:** Official guide, WFME, Accreditation

## Abstract

We are excited to contribute our thoughts and insights to the discussion initiated by Gandomkar et al. in their article on the accreditation system in Iran (Gandomkar et al., BMC Med Educ 23:379, 2023). As individuals who have been directly involved in the process of meta-accreditation and possess a comprehensive understanding of the various stages of Undergraduate Medical Education (UME) accreditation in Iran, we would like to highlight additional points that were identified through a rigorous hermeneutic phenomenology process proposed by Gadamer (Gadamer, Truth and Method, 2013) and offer a complementary point of view to the previous work. By sharing our insights, we hope to contribute to the ongoing discourse surrounding UME accreditation.

There are official bodies for accreditation and post-accreditation monitoring in Iran. The accreditation body is responsible for the official accreditation of undergraduate medical education (UME) programs throughout the country [1]. The post-accreditation monitoring body is responsible for monitoring and controlling the process of accreditation.

In post-accreditation monitoring, we employed Gadamer’s hermeneutic approach, which allowed us to thoroughly examine and interpret the various perspectives and factors influencing the accreditation process, thereby shedding light on the challenges faced [2]. There is a lack of consensus among the experts engaged in the process of accreditation, including internal and external assessors, expert panels, and decision-makers. As a result, there is a need for a unified and coherent interpretation of the standards within the eight areas of accreditation proposed by WFME, contextualized to the Iranian setting. We conducted a post-accreditation monitoring process in order to gain a deeper understanding of this issue and its context within the current developments regarding accreditation in Iran.

To provide clarity for individuals seeking to engage in the accreditation process, we aim to provide further insights and recommendations based on our collective experience and expertise. We propose the development of an official guide that will help settle conflicts and provide a coherent way to interpret the standards.

First, it is important to establish a committee of well-experienced experts to oversee the process. The oversight committee should consist of individuals who possess a strong expertise in the accreditation process, have significant experience in evaluation methodologies, and possess a deep understanding of the specific context of UME programs. Then, it is essential to assign a head for each of the eight areas of accreditation standards to ensure a thorough review. Next, the process should be announced, and external assessors should be assigned in only one of the areas. Additionally, text and content analyses, as well as statistical analyses, should be conducted. This analysis will involve coding and categorization of the extracted data to identify patterns, themes, and potential issues related to standards within each area. Statistical analyses may include descriptive statistics, inferential statistics, and other appropriate statistical techniques to identify any significant variations or deviations from expected norms within the standards of each area. Furthermore, counseling sessions should be set for each area to discuss the standards in relation to the 2020 version of the World Federation for Medical Education (WFME) standards, with the aim of coming to a unified interpretation. Afterward, opinions should be finalized, and negotiation sessions should be arranged in a specialized expert panel committee to confirm the linguistic validity and come to an agreement for the language clarity of each standard. Moreover, each external assessor should be ranked based on the announced criteria, and recommendations for refinement should be provided for those who are weak. Then, a hypothetical medical school should be created to conduct external evaluations and analyze the results, with documents arranged accordingly. Additionally, the content should be refined, and linguistic and medical education experts should be allocated to provide feedback on the clarity of language. Finally, the official guide should be translated into English and sent to the WFME.

The recommendations provided here are not solely based on personal experiences but are also influenced by the employment of official guides in the language assessment field. We believe that the development of an official guide to the UME accreditation process in Iran and other similar countries will help to ensure a unified and coherent interpretation of the standards. However, we should note that the Iranian environment may have specific cultural, social, or contextual factors that need to be considered. These factors might require adaptations or modifications to ensure the guidance aligns with the local context and values. Limited resources, both financial and human, can pose challenges during the creation and application of the official guide. Adequate funding, personnel, and infrastructure must be available to support the development, dissemination, and ongoing implementation of the guide.

## Data Availability

The datasets used and/or analyzed during the current study are available from the corresponding author on reasonable request.
